# E3 Ubiquitin Ligase APC/C^Cdh1^ Negatively Regulates FAH Protein Stability by Promoting Its Polyubiquitination

**DOI:** 10.3390/ijms21228719

**Published:** 2020-11-18

**Authors:** Kamini Kaushal, Sang Hyeon Woo, Apoorvi Tyagi, Dong Ha Kim, Bharathi Suresh, Kye-Seong Kim, Suresh Ramakrishna

**Affiliations:** 1 Graduate School of Biomedical Science and Engineering, Hanyang University, Seoul 04763, Korea; kaminikaushal10@gmail.com (K.K.); tkdgus78902@gmail.com (S.H.W.); apoorvityagi09@gmail.com (A.T.); donha01@naver.com (D.H.K.); bharathi.suri@gmail.com (B.S.); 2 College of Medicine, Hanyang University, Seoul 04763, Korea

**Keywords:** CRISPR/Cas9 knockout, in silico analysis, liver cancer, post-translational modifications, ubiquitin-proteasome system

## Abstract

Fumarylacetoacetate hydrolase (FAH) is the last enzyme in the degradation pathway of the amino acids tyrosine and phenylalanine in mammals that catalyzes the hydrolysis of 4-fumarylacetoacetate into acetoacetate and fumarate. Mutations of the *FAH* gene are associated with hereditary tyrosinemia type I (HT1), resulting in reduced protein stability, misfolding, accelerated degradation and deficiency in functional proteins. Identifying E3 ligases, which are necessary for FAH protein stability and degradation, is essential. In this study, we demonstrated that the FAH protein level is elevated in liver cancer tissues compared to that in normal tissues. Further, we showed that the FAH protein undergoes 26S proteasomal degradation and its protein turnover is regulated by the anaphase-promoting complex/cyclosome-Cdh1 (APC/C)^Cdh1^ E3 ubiquitin ligase complex. APC/C^Cdh1^ acts as a negative stabilizer of FAH protein by promoting FAH polyubiquitination and decreases the half-life of FAH protein. Thus, we envision that Cdh1 might be a key factor in the maintenance of FAH protein level to regulate FAH-mediated physiological functions.

## 1. Introduction

Tyrosine degradation is a crucial five step pathway in animals that breaks down the aromatic amino acids obtained from diet and protein breakdown [1]. In this, the last enzyme used is fumarylacetoacetate hydrolase (fumarylacetoacetate; FAH, E.C. 3.7.1.2), which catalyzes the hydrolysis of fumarylacetoacetic acid into fumaric and acetoacetic acids. FAH is an enzyme encoded by the *FAH* gene located on chromosome 15q25.1 containing 14 exons, spanning over 35 kb of DNA [2,3,4]. It is a cytosolic homodimer with two 46-kDa subunits conformed by a 120-residue N-terminal domain (N-term) and a 300-residue C-terminal domain (C-term). The N-term has an SH3-like fold and plays a regulatory role [5,6]. In contrast, the C-term is defined by a new β-sandwich roll structure that is implicated in metal ion binding and catalysis, participating in intermolecular interactions at the dimer interface [7]. The loss of FAH activity, which is responsible for phenylalanine and tyrosine degradation, would be lethal to humans. Several compounds that accumulate upstream of FAH are toxic alkylating agents that can induce cell apoptosis [8]. Mutations in the *FAH* gene cause type I tyrosinemia (HT1, McKusick; OMIM: 276700), a hereditary metabolic condition characterized by increased tyrosine levels in the blood and urine patients [9]. In the absence of FAH, metabolites such as maleylacetoacetate (MAA), fumarylacetoacetate (FAA) and succinylacetone (SUAC) accumulate during tyrosine degradation. In a mammalian cell assay, FAA has been shown to have mutagenic activity to induce cell cycle arrest at G2/M and apoptosis [8].

Tyrosinemia is characterized by hepatic failure, cirrhosis, renal dysfunction, hepatocarcinoma (HCC) and neurologic crises, in which the liver is the most severely affected organ [10]. Two distinct forms of the disease have been described according to the symptoms and age of onset [10]. The acute condition of tyrosinemia is diagnosed in the first months of life and results in the rapid deterioration of hepatic and renal functions leading to early death due to hepatic failure. In the chronic form, symptoms appear later in childhood and often culminate with the development of hepatocellular carcinoma (HCC). Thus, investigating the regulatory properties of FAH protein is necessary to understand the pathophysiological mechanisms in tyrosinemia and to delineate novel therapeutic strategies. More than 100 mutations were reported in the *FAH* gene causing HT1; among them, most of the mutations lead to the destabilization of FAH protein, resulting in aggregation of the protein [11]. FAH is also linked with ubiquitin at numerous lysine residues as per the information in the PhosphoSitePlus (www.phosphosite.org) database. However, there are no reports to our knowledge about FAH protein ubiquitination and degradation or types of ubiquitin linkages associated with FAH protein and its cellular activities. Considering the reduced activity and deficiency of FAH observed in HT1 [12], research focusing on FAH protein ubiquitination and factors affecting its protein turnover is essential.

Post-translational modifications (PTMs), such as ubiquitination and deubiquitination, play a significant role in many cellular processes, including proliferation and differentiation, by modulating the activities of target proteins and their related enzyme activities in cells [13,14]. Ubiquitination is a post-translational modification that regulates the protein turnover and stabilization of any substrate by marking it for degradation by the 26S proteasome pathway. The ubiquitin proteasomal system (UPS) is regulated mainly by ubiquitin-activating enzyme (E1), ubiquitin conjugases (E2) and ubiquitin ligases (E3) [15,16].

E3 ligases are a component of UPS that works in conjunction with an E1 ubiquitin-activating enzyme and an E2 ubiquitin-conjugating enzyme to transfer ubiquitin to a specific substrate protein. Ubiquitin is a highly conserved, small protein having 76 amino acids that determine the fate of its substrate proteins. Polyubiquitination of proteins marks their degradation via the 26S proteasomal pathway. The anaphase-promoting complex or cyclosome (APC/C) is a multi-subunit cullin-RING E3 ubiquitin ligase that catalyzes the ubiquitination of crucial molecules to regulate cell cycle progression [17]. Cdc20 homologue-1 (Cdh1), as one of the two coactivators of APC, directly interacts with the conserved motifs on the substrates, thereby recruiting them to the APC core [18,19]. On the other hand, cyclin-dependent kinases (CDKs) separate Cdh1 from the APC core through Cdh1 phosphorylation, thereby terminating the activity of APC/C^Cdh1^ to ensure G1/S transition [20,21].

In this study, we analyzed the protein expression levels of FAH in normal and liver tumor tissues. Then, we identified that Cdh1 governed FAH protein destabilization by FAH ubiquitination and subsequent degradation in an APC-dependent manner. The ubiquitin ligase APC/C^Cdh1^ acts as a negative stabilizer of FAH protein by promoting FAH polyubiquitination. Finally, Cdh1 interacts with FAH and exhibits a negative influence on the FAH protein half-life.

## 2. Results

### 2.1. FAH Protein Expression in Human HCC Tumor Tissues

**Figure 1:**
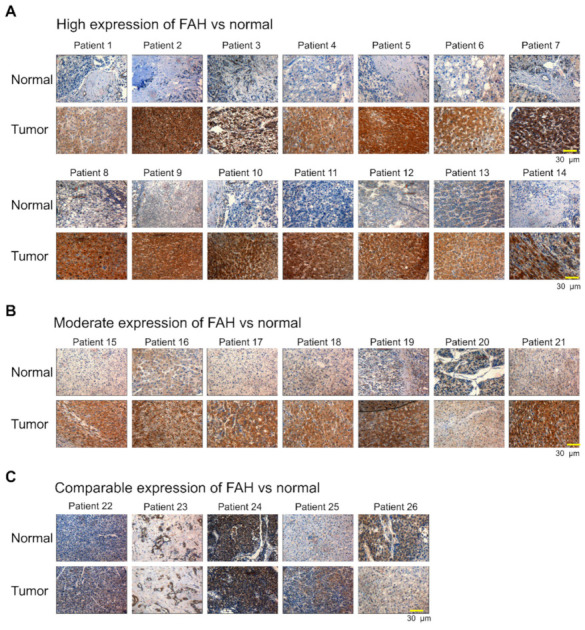
Fumarylacetoacetate hydrolase (FAH) protein expression in human hepatocellular carcinoma (HCC) tumor tissues. (**A**) Representative immunohistochemical staining of endogenous FAH in 14 patient-derived normal and tumor tissues showing high expression of FAH in tumor samples. (**B**) Representative immunohistochemical staining of endogenous FAH in 7 patient-derived normal and tumor tissues showing moderate expression of FAH in tumor samples. (**C**) Representative immunohistochemical staining of endogenous FAH in 5 patient-derived normal and tumor tissues showing comparable expression of FAH in tumor samples (scale bar = 30 µm).

Aberrations in the *FAH* gene cause HT1, resulting in reduced activity and subsequently leading to hepatic failure, cirrhosis, renal dysfunction and HCC [12]. Thus, we wished to investigate the mRNA and protein expression levels of FAH in human tissues using a bioinformatics database. The Human Protein Atlas database suggested that the protein expression of FAH was detected in limited organs; high expression levels were confined to the liver and renal tissues (Supplementary Figure S1A). Additionally, several datasets such as the consensus dataset, HPA dataset, GTEx dataset and FANTOM5 dataset showed high mRNA expression of the *FAH* gene only in the liver and adipose tissue (Figure S1B–E). These in silico data demonstrate that the expression pattern of FAH is dominant in the liver. Thus, we wished to analyze the expression of FAH in liver cancer and normal tissues. To this end, we clinically examined liver cancer and normal tissues from 26 liver cancer patients obtained from the ISU ABXIS cohort. Our results showed that out of 26 liver cancer patients, 14 patients showed significantly high expression of FAH in tumor tissues when compared to the normal tissues (Figure 1A). 7 patients showed moderate expression of FAH in tumor tissues compared to that in normal tissues (Figure 1B) and 5 patients showed non-significant changes in the expression of FAH protein between tumor and normal tissues (Figure 1C). Our results indicate that the expression of FAH protein is elevated in patients suffering from HCC. To support our results, we analyzed the expression levels of FAH using The Cancer Genome Atlas (TCGA) data in liver hepatocellular carcinoma (LIHC), for which normal-tumor matched RNAseq expression data were available. The results showed slight upregulation of the *FAH* gene in tumor tissue when compared with normal tissues (Supplementary Figure S2). Considered together, our results showed that the expression of FAH protein but not the mRNA expression of FAH, is significantly upregulated in HCC, indicating the importance of factors affecting FAH protein turnover.

### 2.2. FAH Undergoes Proteolysis through the 26S Proteasomal Pathway

**Figure 2:**
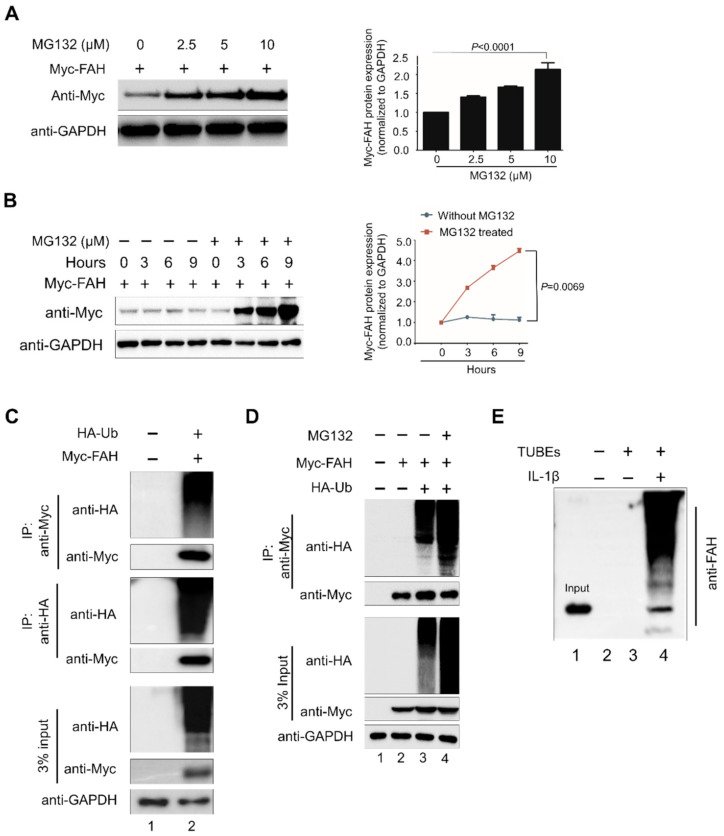
FAH undergoes proteolysis through 26S proteasomal pathway. (**A**) Exogenous Myc-FAH expression upon dose-dependent treatment of MG132 was determined in HEK293 cells through Western blot analysis with suitable antibodies. The Myc-FAH band intensity was estimated by ImageJ software and normalized with Glyceraldehyde 3-phosphase dehydrogenase (GAPDH) as control for graphical representation. Data were presented as the mean and standard deviation of three independent experiments. One-way ANOVA followed by Tukey’s post hoc test was used and *p*-values are indicated. (**B**) HEK293 cells were treated with dimethylsulfoxide as control or MG132 (7.5µM) to check the exogenous Myc-FAH expression through Western blot analysis. The Myc-FAH band intensity was estimated by ImageJ software and normalized with GAPDH for graphical representation. Two-way ANOVA followed by Tukey’s post hoc test was used and *p*-values are indicated. (**C**) Myc-FAH was transfected individually or with HA-ubiquitin into HEK293 cells for immunoprecipitation with Myc antibody followed by immunoblotting with HA or Myc antibody. Reciprocal immunoprecipitation was performed with HA antibody, followed by immunoblotting with HA or Myc antibody. (**D**) Myc-FAH was transfected with HA-ubiquitin and cells were treated with MG132 in HEK293 cells to evaluate the effects on the ubiquitination and proteolysis of Myc-FAH. Cell lysates were immunoprecipitated with Myc antibody followed by immunoblotting with HA or Myc antibody. (**E**) Tandem Ubiquitin Binding Entities (TUBEs) assay for the ubiquitination of FAH proteins were conducted in Human hepatic stellate cells (HHSteCs) in the presence or absence of IL-1β keeping only agarose beads as control. Cell lysates were immunoprecipitated with TUBE antibody followed by immunoblotting with specific anti-FAH antibody. Lane1 is Input, lane 2 is control only agarose beads, lane 3 (without IL-1β) IP with Tubes and immunoblot with anti-FAH antibody and lane 4 (with IL-1β) IP with Tubes and immunoblot with anti-FAH antibody.

To investigate whether FAH is regulated by the UPS degradative pathway, we estimated the FAH protein level in the presence or absence of the proteasomal inhibitor MG132. To this end, Myc-FAH was transfected in HEK293 cells and treated with increasing concentrations of proteasomal inhibitor MG132 for 8 h. We observed that MG132, which blocks 26S proteasome activity, increased FAH protein levels by accumulating proteins in a dose-dependent manner in the cells (Figure 2A). Next we wished to check whether the accumulation of FAH protein occurs due to post-translational modifications or transcriptional regulation. To this end, we performed quantitative real-time RT-PCR (qRT-PCR) for the Myc-FAH transfected samples treated with MG132 in a dose-dependent manner. The results showed that there were no significant changes in the mRNA level of FAH upon MG132 treatment, signifying that the accumulation of FAH protein is mainly due to the blockage of 26S proteasomal degradation system by MG132 (Supplementary Figure S3). To further validate proteasomal regulation of FAH, we performed translation shut off experiment in the presence or absence of MG132 at varied time points. Briefly, HEK293 cells were transfected with constant amount of Myc-FAH and treated with constant amount of either MG132 or DMSO as controls (7.5 μM). These cells were harvested at different time points and subjected to Western blot analysis. Our results indicate a significant increase in the stabilization of FAH protein in MG132 treated cells as compared with the DMSO treated cells (Figure 2B). The increase in FAH protein due to treatment with proteasome inhibitor MG132 led us to speculate that the UPS might be involved in regulating FAH protein turnover. To investigate whether FAH protein undergoes ubiquitination, we co-transfected Myc-tagged-FAH and HA-tagged-ubiquitin in HEK293 cells and performed an immunoprecipitation assay using an anti-Myc antibody followed by immunoblotting with an anti-HA antibody. Our results showed a characteristic high molecular weight smear of polyubiquitin molecules conjugated to FAH protein in the cells co-transfected with the FAH and ubiquitin construct, which indicated that FAH protein undergoes polyubiquitination (Figure 2C, lane 2).

We next examined the effect of MG132 on FAH ubiquitination. The treatment with MG132 of the co-transfected cell cultures for 8 h resulted in the increased accumulation of polyubiquitinated FAH protein (Figure 2D, lane 4) as compared to the untreated samples (Figure 2D, lane 3). Furthermore, to validate whether FAH is undergoing endogenous ubiquitination, we performed TUBEs assay that has high affinity probe for ubiquitinated proteins [22]. This was carried out in HHSteCs in the presence or absence of Interleukin-1 beta (IL-1β), keeping only agarose beads as control. Upon stimulation of IL-1β, FAH protein undergoes rapid ubiquitination (Figure 2E, lane 4) when compared to non-stimulated cells (Figure 2E, lane 3). Thus, our results suggest that FAH protein interacts with ubiquitin molecules and undergoes polyubiquitination through UPS.

### 2.3. E3 Ligase APC/C^Cdh1^ Destabilizes FAH Protein

**Figure 3:**
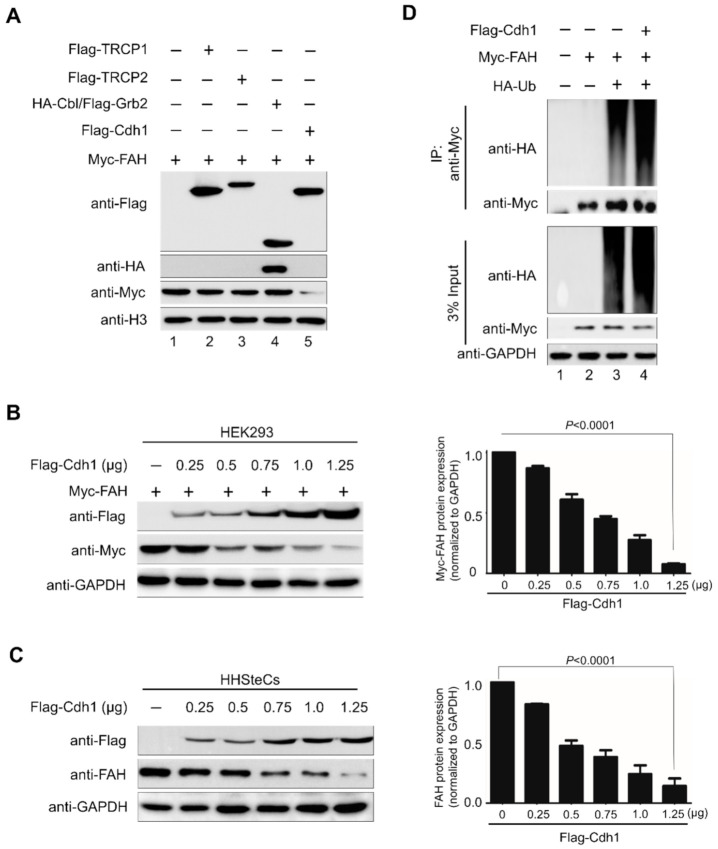
E3 ligase APC/C^Cdh1^ destabilizes FAH protein. (**A**) Screening E3 ligases for FAH. Several ligases were transfected transiently along with Myc-FAH in HEK293 cells and analyzed through Western blotting. (**B**) HEK293 cells were co-transfected with a constant amount of Myc-FAH along with Flag-Cdh1 at increasing concentrations (0, 0.25, 0.5, 0.75, 1.0 and 1.25 µg). The Myc-FAH band intensity was estimated by ImageJ software and normalized with GAPDH for graphical representation. Data were presented as the mean and standard deviation of three independent experiments. One-way ANOVA followed by Tukey’s post hoc test was used and *p*-values are indicated. (**C**) HHSteCs were co-transfected with Flag-Cdh1 at increasing concentrations (0, 0.25, 0.5, 0.75, 1.0 and 1.25 µg) and bands were analyzed by endogenous FAH antibody through Western blotting. The FAH band intensity was estimated by ImageJ software and normalized with GAPDH for graphical representation. Data were presented as the mean and standard deviation of three independent experiments. One-way ANOVA followed by Tukey’s post hoc test was used and *p*-values are indicated. (**D**) Myc-FAH was transfected with HA-ubiquitin along with Flag-Cdh1 in HEK293 cells to see the effect of Cdh1 on the ubiquitination and proteolysis of FAH. Cell lysates were immunoprecipitated with Myc antibody followed by immunoblotting with HA or Myc antibody. GAPDH was used as a loading control.

To analyze the factors regulating the FAH protein levels, we screened a panel of E3 ligases available in our lab that might be involved in the reduction of FAH protein levels by Western blot analysis. Flag-TRCP1, Flag-TRCP2, HA-Cbl/Flag-Grb2 and Flag-Cdh1 were co-transfected with a constant amount of Myc-FAH in HEK293 cells. Among several E3 ligases, Flag-Cdh1 was associated with a significant reduction in FAH protein level (Figure 3A). Next, we transfected Flag-Cdh1 in a dose-dependent manner along with a constant amount of Myc-FAH construct in HEK293 cells. The dose-dependent increase in Cdh1 expression proportionally decreased FAH protein level in a dose-dependent manner, indicating that Cdh1 destabilizes FAH protein (Figure 3B). Similarly, at endogenous levels, we observed that a gradual increase in Cdh1 expression caused a proportional decrease in endogenous FAH expression in HHSteCs (Figure 3C).

Next, we wished to analyze the effect of overexpression of Cdh1 on FAH protein ubiquitination. To this end, we co-transfected HEK293 cells with Myc-FAH, HA-ubiquitin and Flag-Cdh1 and performed immunoprecipitation analysis with anti-Myc antibody followed by immunoblotting with anti-HA antibody. The overexpression of Cdh1 led to increased high molecular weight smear of polyubiquitin chain conjugated with FAH protein (Figure 3D, lane 4) when compared with the absence of Cdh1 on FAH polyubiquitination (Figure 3D, lane 3). Thus, our results signify that APC/C^Cdh1^ is an E3 ligase responsible for the destabilization and protein degradation of FAH protein.

### 2.4. APC/C^Cdh1^ Acts as a Negative Regulator of FAH Protein Stability

**Figure 4:**
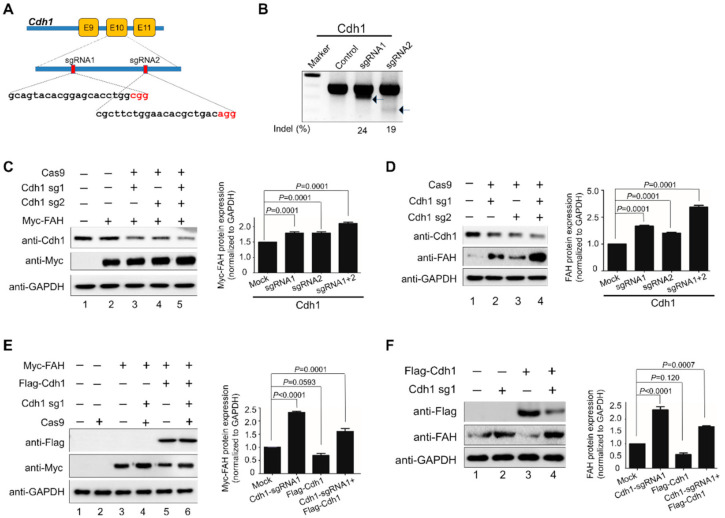
APC/C^Cdh1^ is a negative regulator of FAH protein stability. (**A**) Schematic of the RNA-guided engineered nuclease targeting the human Cdh1 gene with designed sgRNA1 and sgRNA2 that target sequences in exon 10. Yellow boxes represent exons. Red box indicates the positions of sgRNAs targeting the top strand. Target sequences are represented in black and PAM sequences are represented in red. (**B**) T7E1 assays were performed in HEK293 cells to determine the cleavage efficiency of sgRNA-1 and sgRNA-2 by transfecting Cas9, sgRNA-1 and sgRNA-2. The samples were loaded in 2% agarose gel. The two bands in the sgRNA-1 and sgRNA-2 results indicate the cleaved size of DNA and are indicated by arrows along with the indel percentages measured by band intensity using ImageJ software. Untransfected HEK293 cells were used as a control. The marker is shown for size reference. (**C**) HEK293 cells were transiently transfected with sgRNA-1 and sgRNA-2 targeting Cdh1 along with ectopically expressed Myc-FAH to check exogenous protein levels. (**D**) HHSteCs were transfected with sgRNA-1 and sgRNA-2 targeting Cdh1 to check endogenous FAH protein levels using FAH specific antibody and endogenous Cdh1 was detected using anti-Cdh1 antibody. (**E**) Reconstitution effect of Cdh1 on exogenous FAH protein level in Cdh1-depleted HEK293 cells. (**F**) Reconstitution effect of Cdh1 on endogenous FAH protein level in Cdh1-depleted HHSteCs. The FAH band intensity for (**C**–**F**) was estimated by ImageJ software and normalized with GAPDH for graphical representation. Data were presented as the mean and standard deviation of three independent experiments. One-way ANOVA followed by Tukey’s post hoc test was used and *p*-values are indicated.

To empathize the physiological role of APC/C^Cdh1^ in regulating the *FAH* gene, we designed two sgRNAs specific to the exon 10 region of Cdh1 by using a clustered, regularly interspaced, short palindromic repeats/CRISPR-associated-9 (*CRISPR/Cas9*) knockout system through the Genetic Perturbation Platform (GPP) sgRNA designer (www.broadinstitute.org) website (Figure 4A). The gene disruption efficiency of the sgRNAs was estimated through the high indel percentage obtained from the T7E1 assay and Western blot analysis. The efficiency of sgRNA-1 showed high indel percentage than sgRNA-2 targeting the Cdh1 gene (Figure 4B). Then, we assessed the effect of sgRNA-1 and sgRNA-2 targeting Cdh1 on expression of FAH exogenously. The results showed that the sgRNA-1 targeting Cdh1 upregulated FAH protein when compared to mock (Figure 4C, lane 3). However, the combined effect of both sgRNA-1 and sgRNA-2 showed high upregulation of FAH protein when compared with sgRNA-1 alone targeting the Cdh1 gene (Figure 4C, lane 5). A similar experiment was conducted in HHSteCs in which the sgRNA-1 expression was upregulated in FAH protein (Figure 4D, lane 2) and the combined effect of both sgRNA-1 and sgRNA-2 showed an increase in the endogenous FAH protein level (Figure 4D, lane 4). To validate the specificity of Cdh1 on the FAH protein stabilization, Cdh1-depleted cells were reconstituted with Cdh1 and the expression of FAH was analyzed. Our results showed stabilization of FAH protein in Cdh1 depleted cells when compared with mock (Figure 4E, lane 4). However, FAH protein stabilization was reversed when Cdh1-depleted cells were reconstituted with ectopically expressed Flag-Cdh1 (Figure 4E, lane 6). Similarly, endogenous FAH protein stabilization effect was reversed when Cdh1-depleted cells were reconstituted with ectopically expressed Flag-Cdh1 in HHSteCs (Figure 4F, lane 4) when compared with endogenous expression of FAH in Cdh1-depleted cells (Figure 4F, lane 2). Our results prove that Cdh1 is a specific E3 ligase for FAH protein.

### 2.5. APC/C^Cdh1^ Co-Localizes and Interacts with FAH

**Figure 5:**
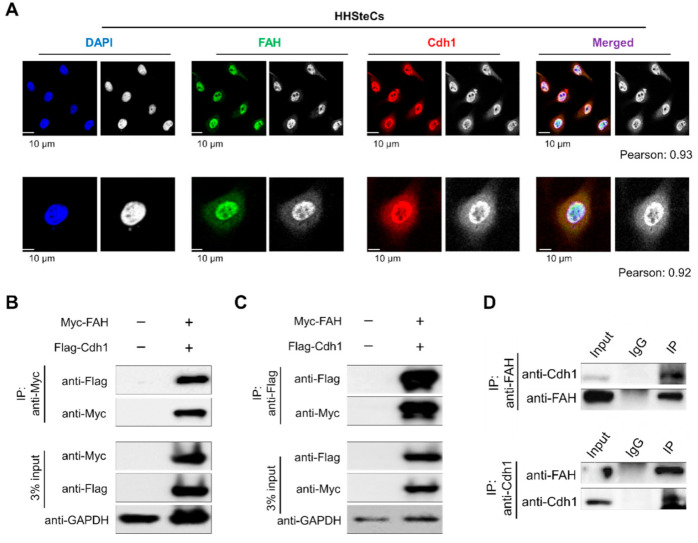
APC/C^Cdh1^ co-localizes and interacts with FAH. (**A**) Co-localization of FAH and Cdh1 was performed in HHSteCs by immunostaining with specific antibodies. DAPI was used for nuclear staining (scale bar: 10 µm) and the quantification for colocalization coefficients were done using Fiji/ImageJ software. To quantify the degree of co-localization in between the fluorochromes, the Pearson’s correlation coefficient (r) was used. The classical Pearson coefficient of the pixel-intensity correlation of colocalization obtained from the coloc2 analysis were mentioned. (**B**) Myc-tagged FAH and Flag-tagged Cdh1 were co-transfected into HEK293 cells. Samples were immunoprecipitated using anti-Myc and immunoblotted using anti-Flag antibodies. GAPDH was used as the loading control. (**C**) Myc-tagged FAH and Flag-tagged Cdh1 were co-transfected into HEK293 cells. Samples were immunoprecipitated using anti-Flag and immunoblotted using anti-Myc antibodies. GAPDH was used as the loading control. (**D**) Endogenous binding between FAH and Cdh1 proteins was performed in HHSteCs. Cell lysates from HHSteCs were immunoprecipitated with specific FAH or Cdh1 antibodies and immunoblotted with FAH or Cdh1 antibodies.

To investigate the cellular localization of Cdh1 and FAH, we performed immunofluorescent staining of endogenous Cdh1 and FAH protein together in HHSteCs. Our results showed that Cdh1 and FAH protein are co-localized in both the nucleus and the cytoplasm, predominantly in the nucleus (Figure 5A). Next, we analyzed the physical interaction between the two proteins *in vivo*. To this end, immunoprecipitation was performed by co-transfecting with Myc-FAH and Flag-Cdh1 in HEK293 cells. Myc-FAH could co-immunoprecipitate with the Flag-Cdh1 and vice versa (Figure 5B,C). Additionally, endogenous interaction between FAH and Cdh1 was demonstrated by co-immunoprecipitation assay using a specific anti-FAH antibody that could co-precipitate endogenous Cdh1 (Figure 5D, upper panel). In a reciprocal immunoprecipitation assay, anti-Cdh1 antibody co-precipitated endogenous FAH (Figure 5D, lower panel). Thus, our results signify that Cdh1 and FAH interact with each other at both exogenous and endogenous levels.

### 2.6. APC/C^Cdh1^ Promotes FAH Polyubiquitination

**Figure 6:**
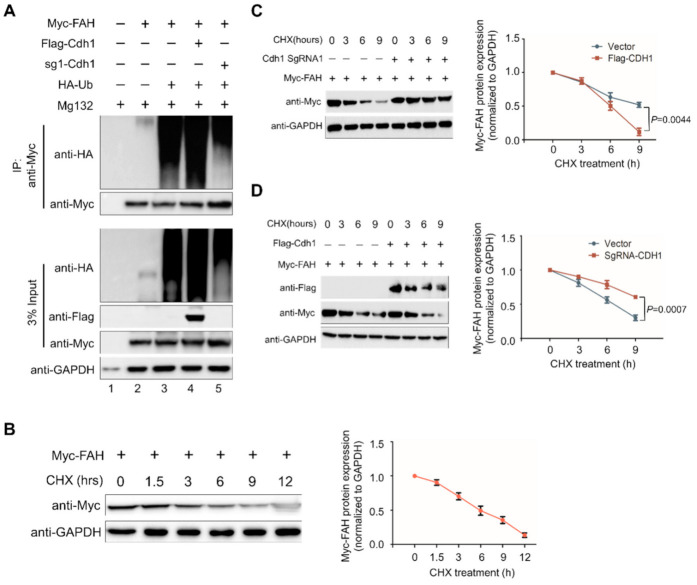
APC/C^Cdh1^ promotes FAH polyubiquitination and decreases FAH protein half-life. (**A**) Flag-Cdh1, sgRNA-1-Cdh1 and HA-ubiquitin were co-transfected into HEK293 cells with Myc-FAH and treated with MG132 (5 µM) for 8 h. The cells were immunoprecipitated with Myc-antibody followed by immunoblotting with HA and Myc antibodies and the polyubiquitination of FAH was examined. (**B**) Determination of FAH protein half-life. HEK293 cells were transfected with Myc-FAH and control vector and treated with cycloheximide (CHX) (150 µg/mL). Cells were harvested at specific time intervals and analyzed by Western blotting with the Myc antibody indicated. (**C**) FAH protein half-life is extended upon the transfection of sgRNA targeting Cdh1. HEK293 cells were transfected with the control vector and sgRNA-Cdh1, treated with CHX and analyzed by Western blotting. (**D**) FAH protein half-life is reduced upon transfection of Flag-Cdh1. HEK293 cells were transfected with control vector and Flag-Cdh1 and treated with CHX and expression levels were analyzed by Western blotting. GAPDH was used as a loading control. The FAH band intensities for (**B**–**D**) were estimated by ImageJ software and normalized with GAPDH for graphical representation. Data were presented as the mean and standard deviation of three independent experiments. Two way ANOVA followed by Tukey’s post hoc test was used for comparison between two groups and the *p*-values are indicated.

To analyze the effect of Cdh1 on the ubiquitination status of FAH protein, we overexpressed Cdh1 or knocked out Cdh1 along with HA-ubiquitin construct in HEK293 cells. Transfected cells were treated with MG132 for 8h and immunoprecipitation was carried out using anti-Myc, followed by immunoblotting with respective antibodies. An intense ubiquitin smear was observed in cells co-transfected with Flag-Cdh1 along with HA-ubiquitin and Myc-FAH (Figure 6, lane 4) when compared with cells co-transfected with HA-ubiquitin and Myc-FAH (Figure 6, lane 3). In contrast, the ubiquitin smear was reduced in the cells depleted with Cdh1 when compared with cells cotransfected with HA-ubiquitin and Myc-FAH (Figure 6, lane 5). Thus, the aforementioned data indicate that APC/C^Cdh1^ promotes the conjugation of polyubiquitin molecules to FAH protein, leading to rapid degradation.

### 2.7. APC/C^Cdh1^ Regulates FAH Protein Half-Life

We wished to check the half-life of FAH protein and the effect of the overexpression of Cdh1 on FAH protein half-life. To analyze FAH protein turnover in the presence and absence of Cdh1, we performed cycloheximide assay in HEK293 cells transfected with Flag-Cdh1 or sgRNA-1 targeting Cdh1 along with ectopically expressed Myc-FAH. Our results showed that the half-life of FAH is about 6 h (Figure 6B). Interestingly, the knockout of Cdh1 led to the extension of FAH protein half-life when compared with samples transfected with Myc-FAH alone (Figure 6C). In contrast, Myc-FAH protein half-life was significantly reduced in the presence of Cdh1 when compared with samples transfected with Myc-FAH alone (Figure 6D). Thus, the depletion of Cdh1 is responsible for the extension of FAH protein half-life.

## 3. Discussion

FAH is the final enzyme within the tyrosine catabolism pathway that catalyzes the hydrolysis of FAA into fumarate and acetoacetate as the last step in phenylalanine and tyrosine degradation. In 1932, Grace Medes found 4-hydroxyphenylpyruvate within the urine of a 49-year-old man and termed it as “tyrosinosis” [23]. Later, in the 1960s, the condition was alluded to as HT1, an outcome of FAH deficiency [24,25,26]. Deficiency of FAH leads to the accumulation of upstream metabolites such as FAA and MAA ultimately converting into SUAC, of which FAA and SUAC are known to be both genotoxic and carcinogenic [27]. HT1 pathogenicity is mostly indefinite but a few missense mutations in the *FAH* gene may influence catalytic activity, protein stability, protein homeostasis and monomer-dimer equilibrium [11].

For the remedial cure for HT1, dietary restriction in phenylalanine and tyrosine, liver transplantation and an inhibitor of 4-hydroxyphenylpyruvic acid dioxygenase (nitisinone) also known as NTBC (2-[2-nitro-4-(trifluoromethyl)benzoyl] cyclohexane-1,3-dione) are the known therapies but they have their limitations such as side effects and long-term complications for the patients [28]. Thus, HT1 requires substitute therapeutic intercession lines that would suggest less harmful side impacts. Mutated proteins are susceptible for misfolding which is corrected by protein control systems such as molecular chaperones and UPS. Molecular chaperones are proteins that guide the assembly and conformational folding of macromolecular structures or *vice versa*. These are heat shock proteins (HSPs) produced by cells under stressful conditions that stabilize new proteins to ensure proper folding or refold damaged proteins [29]. Additionally, there are several E3 ligases that intimately coordinate with molecular chaperones for protein refolding or the degrading of misfolded proteins [30]. Quantitative protein interaction analysis suggests that more than 30% of all human E3 ligases interact with Hsp90 [31].

To date, studies related to PTMs and their regulation of FAH protein function have not yet been reported. However, the only described FAH superfamily members in the eukaryotic kingdom are fumarylacetoacetate hydrolase domain-containing proteins (FAHDs) 1 and 2, which have been a focus of recent work in aging research [32]. The high-throughput proteomics analysis suggests that the most probable interaction partners of FAHD1 are carnitine palmitoyltransferase 2 (CPT2), clustered mitochondria homolog (CLUH), NADH dependent ubiquinone oxidoreductase subunit S6 (NDUFS6), polyribonucleotide nucleotidyltransferase 1 (PNPT1) and putative ubiquitin protein ligase E3 component n-recognin 3 (UBR3). Putative UBR3 is an E3 ubiquitin-protein ligase, which is a component of the *N*-end rule pathway, leading to ubiquitination and the subsequent degradation of its target proteins [33,34]. Thus, we were curious to know about the specific E3 ligases that regulate the ubiquitination and degradation of FAH at the physiological and pathological levels.

APC/C^Cdh1^ catalyzes the direct attachment of ubiquitin monomers to the protein target at multiple lysine sites in a random pattern. It has been reported that the multiple monoubiquitination of cyclin B1 is triggered by APC, which provides a signal for proteolysis [35]. Previous work has revealed that APC/C^Cdh1^ regulates craniofacial development through the monoubiquitination of Gsc [36]. Besides monoubiquitination, APC associates with two E2 conjugating enzymes, Ube2S and Ube2C, to induce K11-linked ubiquitin chains on the substrates, thus orchestrating the progression of cells through mitosis [37]. In Alzheimer’s disease and stroke pathogenesis, APC/C^Cdh1^ but not Cdc20, is expressed in post-mitotic mammalian neurons and the inactivation of Cdh1 by phosphorylation results in the accumulation of cyclin B1 [38]. Thus, we predicted that finding an E3 ligase governing FAH ubiquitination might serve as a therapeutic target in HT1.

Hepatocellular carcinoma is one of the most prevalent causes of cancer-related deaths worldwide and is commonly associated with viral hepatitis, chronic exposure to toxins and hereditary liver diseases [39]. FAA is a thiol-reacting and organelle/mitotic spindle-disturbing agent with mutagenic activities. Accumulation of the metabolite FAA is the hallmark of HT1. Hence, liver injury in HT1-deficient organisms has been mainly attributed to the immediate toxicity of the metabolite [40]. Thus, these studies prompted us to investigate the expression of FAH in HCC patient tissues. By using in silico analysis, we found that FAH protein expression is restricted to the liver and adipose tissues (Figure S1). Additionally, *FAH* was upregulated in LIHC (Figure S2). Furthermore, Immunohistochemistry (IHC) staining for FAH in liver cancer tissues showed very high expression levels in most of the cancer patients when compared with normal tissues (Figure 1). Thus, we initiated our research on whether FAH protein is regulated by 26S proteasome and on screening the E3 ligases responsible for FAH protein turnover.

Here, we delineate the role of APC/C^Cdh1^ in maintaining FAH enzyme levels in cells. For the first time, we demonstrated that FAH protein indeed undergoes 26S proteasomal degradation (Figure 2). Furthermore, the overexpression of Cdh1 promotes FAH protein ubiquitination and destabilizes it in a dose-dependent manner (Figure 3). To support our data for identifying Cdh1 as a specific E3 ligase, *CRISPR/Cas9*-mediated knockout of the Cdh1 gene showed a stabilizing effect on FAH protein levels by preventing FAH protein degradation (Figure 4). We further demonstrated that Cdh1 and FAH protein co-localize in both the cytoplasm and the nucleus. Additionally, we showed that both proteins interact at exogenous and endogenous levels (Figure 5). As a functional consequence of interaction, Cdh1 promotes FAH protein polyubiquitination and subsequently decreases the half-life of FAH protein (Figure 6). Thus, our data signifies that the depletion of Cdh1 results in the stabilization of FAH protein by preventing its ubiquitination and signifies the importance of E3 ligase in FAH-mediated metabolism. Overall, there should be a perfect balance between E3 ligases which signals for protein degradation and deubiquitinating enzymes which prevents protein degradation to regulate appropriate level of FAH protein in the cells for maintaining cellular homeostasis.

In conclusion, our study shows the first attempt to identify an E3 ligase that regulates the stability and turnover of FAH. Furthermore, we identified APC/C^Cdh1^ as an E3 ligase that promotes the polyubiquitination of FAH, resulting in the destabilization of FAH protein. Thus, we envision that Cdh1 might be a potential therapeutic target to regulate FAH-mediated physiological disorders.

## 4. Materials and Methods

### 4.1. Plasmids

A mammalian expression vector encoding GFP-tagged FAH was kindly provided by Prof. Oscar Millet (Protein Stability and Inherited Disease Laboratory, Bizkaia, Spain). The GFP-FAH was further subcloned into the pcDNA 3.1 6XMyc-vector. Flag-tagged ubiquitin, Flag-tagged Grb2 and HA-tagged Cbl were kindly provided by Prof. Yun Soo Bae (Ewha University, Seoul, South Korea). Flag-tagged TRCP1, Flag-tagged TRCP2 and Flag-tagged Cdh1 were kindly provided by Prof. Zhao Qi Wang (Leibniz Institute on Aging—Fritz Lipmann Institute, Jena, Germany) and HA-tagged ubiquitin (Cat no. #18712) were purchased from Addgene.

### 4.2. Antibodies and Reagents

Mouse monoclonal antibodies against HA (sc-7392, 1:1000), Flag (Anti-DDDDK-tag, M185-3L, 1:1000) (MBL Life Science, Woburn, MA, USA), Histone H3 (CST-9751, 1:1000), GAPDH (sc-32233, 1:1000), ubiquitin (sc-8017, 1:1000) and normal mouse IgG (sc-2025, 1:1000) were purchased from Santa Cruz Biotech, Dallas, TX, USA; Histone H3 (CST-9751, 1:1000) were purchased from Cell Signaling Technology, Danvers, MA USA, rabbit polyclonal antibodies against FAH (LS-C165918, 1:1000) and FZR1/Cdh1 (Cat no. #34-2000, Invitrogen, 1:1000) were purchased from LS-Bio, Seattle, WA, USA and Invitrogen, Carlsbad, CA, USA, respectively. In addition, 488/594-conjugated secondary antibodies (Cat no. #A21207, Cat no. #A21203, 1:200) (Life Technologies, Carlsbad, CA, USA) were used. Protein A/G Plus Agarose beads (sc-2003, Santa Cruz Biotech, Dallas, TX, USA); protease inhibitor cocktail (Cat no. #B14012, Bimake.com, Houston, TX, USA), protein translation inhibitor cycloheximide (CHX; Cat no. #239765, Merck, Kenilworth, NJ, USA), RIPA buffer (Cat no. #R2002, Bioseong), Protein 5X sample buffer (Cat no. #EBA-1052, ELPIS BIOTECH, Taejon, Korea), proteasomal inhibitor MG132 (Cat no. #S2619, Selleckchem, Houston, TX, USA) and TUBE 2 (Cat no. #UM402, Life Sensors, Malvern, PA, USA) were also used.

### 4.3. Cas9 and sgRNA Constructs

To screen the single-guide RNAs (sgRNAs), we used a plasmid encoding Cas9-2a-mRFP-2a-PAC (puromycin N-acetyl-transferase, a puromycin resistance gene) and a plasmid encoding sgRNAs, which were purchased from Toolgen (Seoul, South Korea). The sgRNA target sequences were based on bioinformatics tools (www.broadinstitute.org) and cloned into the vectors described previously [41]. Briefly, oligonucleotides containing each target sequence were synthesized (Bioneer, Seoul, South Korea) and T4 polynucleotide kinase was used to add terminal phosphates to the annealed oligonucleotides (BioRad, Hercules, CA, USA). The vector was digested with *BsaI* restriction enzyme and ligated with the annealed oligonucleotides. Oligonucleotide sequences are listed in the Supplementary Table S1.

### 4.4. Cell Culture and Transfection

Human embryonic kidney (HEK293) cells were cultured in DMEM (GIBCO BRL, Rockville, MD, USA) supplemented with 10% fetal bovine serum (FBS; GIBCO BRL, Rockville, MD, USA) and 1% penicillin and streptomycin (GIBCO BRL, Rockville, MD, USA) at 37 °C in a humidified atmosphere with 5% CO_2_. Human hepatic stellate cells (HHSteCs) were kindly provided by Prof. Yun Soo Bae (Ewha University, Seoul, South Korea) and cultured in DMEM supplemented with 20% FBS and 1% penicillin and streptomycin at 37 °C in a humidified atmosphere with 5% CO_2_. The cells were passaged every 3–4 days depending on cell confluence. For all exogenous and endogenous experiments, we used HEK293 cells and HHSteCs, respectively.

For transient transfection, HEK293 cells and HHSteCs were transfected with plasmids using polyethyleneimine (PEI; Polysciences, Warrington, PA, USA) according to the manufacturer’s protocol. HHSteCs were transfected with sgRNA and Cas9 targeting *Cdh1* for the knockout of *Cdh1*. The transfected cells were selected the next day by incubating them with puromycin (1 μg/mL) for 2 days and were then passaged until further use. For the endogenous binding and ubiquitination, the cells were treated with 20 μM MG132 for 8 h before harvesting.

### 4.5. Real-Time PCR

Total RNA was isolated using Trizol reagent (Favorgen, Kaohsiung, Taiwan). RNA pellets were suspended in 50 μL diethylpyrocarbonate (DEPC) water and the RNA concentration was determined. Total mRNA was reverse transcribed into cDNA using the SuperScript III First-Strand Synthesis System (Life Technologies) with Oligo (dt) primers. Quantitative PCR was performed using Fast SYBR Green master mix (Life Technologies) and a StepOnePlus Real-Time PCR System (Life Technologies, USA) with FAH-targeting primers (5′-TCGGAAGTGTGCATTCATCTC-3′ and 5′ TCAACGCATTCTCCTTGTCC-3′) and GAPDH targeting primers (5′-CATGTTCGTCATGGGTGTGAACCA-3′ and 5′-AGTGATGGCATGGACTGTGGTCAT-3′).

### 4.6. T7 Endonuclease I (T7E1) Assay

T7E1 assay was performed as previously described [42]. Isolation of genomic DNA was performed using DNeasy Blood & Tissue kits (Qiagen, Hilden, Germany) according to the manufacturer’s instructions. The region of DNA containing the nuclease target site was PCR-amplified using Hemi-nested primers. The oligonucleotide sequence information to obtain PCR amplicons for the T7E1 assay and the expected cleavage sizes after the T7E1 assay are mentioned in Supplementary Tables S2 and S3. Amplicons were then denatured by heating and annealed to form heteroduplex DNA, which was treated with 5 units of T7E1 (New England Biolabs, Ipswich, MA, USA) for 20 min at 37 °C and analyzed using 2% agarose gel electrophoresis. Mutation frequencies were calculated based on band intensity using ImageJ software and the following equation: mutation frequency (%) = 100 × (1 − [1 − fraction cleaved] 1/2), where fraction cleaved was the total relative density of the cleavage bands divided by the sum of the relative density of cleavage and uncut bands.

### 4.7. Immunoprecipitation and Immunoblotting

For exogenous ubiquitination and immunoprecipitation assays, HEK293 cells after 48 h of transfection with their respective constructs were harvested and lysed in buffer B containing 50 mM Tris-HCl (pH 7.6), 150 mM NaCl, 1 mM EDTA, 1% Triton X-100 and 1 mM PMSF. About 2–3 mg of cell lysates were incubated with the respective antibodies at 4 °C overnight followed by immunoprecipitation with 25 μL of protein agarose beads at 4 °C for 2–3 h. The beads were washed with cell lysis buffer containing 150 mM sodium chloride, 1% triton X-100, 1% sodium deoxycholate, 0.1% SDS, 50 mM Tris-HCl, pH 7.5 and 2 mM EDTA and eluted in eluted in 2X denaturing protein sample buffer containing 312.5 mM Tris-HCl (pH 6.8), 50% glycerol, 5% SDS, 5% β-mercaptoethanol, 0.05% bromophenol blue and subjected to boiling at 95 °C–100 °C for 5 min. These samples were then analyzed by Western blot using the ChemiDoc Imaging System. For endogenous immunoprecipitation assays, HHSteCs were treated with proteasomal inhibitor MG132 for 8 h before harvesting. About 5 mg of lysate were incubated with the respective antibodies and detected using Western blot analysis, where 3% of the samples were used to identify immunoprecipitation efficiency as whole cell lysate. Mouse IgG (ab-99697) and rabbit IgG (CST- 58802S) light chain-specific secondary antibodies were used to prevent interference from heavy and light immunoglobulin chains in the binding assay.

### 4.8. TUBEs Ubiquitination Assay

The endogenous ubiquitination of FAH was validated in HHsteCs using the Tandem Ubiquitin Binding Entities (TUBEs) ubiquitination assay. Cells treated with IL-1β (5 ng/mL, Sigma-Aldrich, St. Louis, MO, USA) were harvested and lysed and the poly Ub chains were captured from the cell lysate using TUBEs. Then, the samples were subjected to western blotting with the indicated antibodies.

### 4.9. Immunofluorescence Microscopy

Around 10,000 HHSteCs were seeded in 4-well culture dishes and incubated at 37 °C in a humidified atmosphere with 5% CO_2_ for two days. After PBS wash, the cells were fixed for 20 min in 4% paraformaldehyde (PFA), permeabilized with 0.1% Triton X-100 in PBS for 5–10 min and incubated with suitable primary antibodies diluted in bovine serum albumin (BSA) at 4 °C overnight. The next day, after PBS, the slides were incubated with 1 μg/mL Alexa Fluor 488/594-conjugated secondary antibodies and counterstained using VECTASHIELD anti-fade mounting medium with DAPI for staining. The stained cells were captured using a Leica fluorescence microscope (Leica, DM 5000 B; Leica CTR 5000; Wetzlar, Germany). These images were imported into Fiji version (http://fiji.sc) of the free image processing software ImageJ. Fiji contains pre-installed plugins such as coloc2 which calculates several colocalization parameters such as Pearson coefficient, Manders correlation and others, which is based on the pixel-intensity correlation. Values close to zero represent weak association while values between 0.5-1 represent biological meaningful association [43]. The Pearson coefficient values of the pixel-intensity correlation of colocalization between Cdh1 and FAH protein were calculated.

### 4.10. Expression and Survival Analysis Based on The Cancer Genome Atlas Data

The Cancer Genome Atlas (TCGA) expression data sets were downloaded from the University of California Santa Cruz (UCSC) Xena website (https://xenabrowser.net/) as processed data (level 3). For both normal and tumor patient samples, gene expression comparison was analyzed by a paired t-test. Kaplan-Meier survival analysis was performed using the R ‘survival’ package version 2.4 for overall survival (OS) and was estimated as the time from diagnosis to death from any cause. For the analysis of statistical significance between the expression levels of the gene of interest in the top and bottom tertile groups of patients, log-rank tests were performed. A *p*-value less than 0.05 was considered statistically significant.

### 4.11. Immunohistochemistry

Clinical samples for normal and liver cancer tissues were obtained from AccMax Array Inc. (Cat no. #A304, ISU Abxis Co., Seoul, Korea). The formalin-fixed, paraffin-embedded (FFPE) tissue specimens were deparaffinized and incubated with anti-FAH (1:50) or anti-Cdh1 (1:200) according to a protocol described previously [44]. The slides were counterstained with hematoxylin, dehydrated and mounted. The stained slides were captured using a Leica DM5000 B.

### 4.12. Statistics

For the statistical analyses, GraphPad Prism 9 (GraphPad Software, Inc. CA, USA) was used and data are presented as means ± standard deviations of three independent experiments. One-way analysis of variance (ANOVA) was used to analyze the data and multiple comparisons among the groups were performed by Tukey’s post hoc test. For comparison between two groups, two-way ANOVA was used to analyze the data. A *p*-value less than 0.05 was considered statistically significant.

## Supplementary Materials

Supplementary materials can be found at https://www.mdpi.com/1422-0067/21/22/8719/s1.
