# Supplementary material for: E3 Ubiquitin Ligase APC/CCdh1 Negatively Regulates FAH Protein Stability by Promoting Its Polyubiquitination
Source: Int J Mol Sci. 2020 Nov 18;21(22):8719. doi: 10.3390/ijms21228719 (PMC7699203; doi:10.3390/ijms21228719)
Supplement: Supplementary file 1 [file ijms-21-08719-s001.pdf]

E3 ubiquitin ligase APC/C^{Cdh1} negatively regulates FAH protein stability by promoting its polyubiquitination

Kamini Kaushal[1†], Sang Hyeon Woo[1†], Apoorvi Tyagi[1], Dong Ha Kim[1], Bharathi Suresh[1], Kye-Seong Kim[1,2]* and Suresh Ramakrishna[1,2]*

[1]Graduate School of Biomedical Science and Engineering, Hanyang University, Seoul 04763, South Korea

[2]College of Medicine, Hanyang University, Seoul 04763, South Korea

† These authors have contributed equally: Kamini Kaushal[1†] and Sang Hyeon Woo[1†]

***Corresponding authors**

SR (E-mail: suri28@hanyang.ac.kr, suresh.ramakris@gmail.com);

KS (E-mail: ks66kim@hanyang.ac.kr)

Table of Contents

Figure S1. Comparison of FAH protein expression and mRNA expression in different tissue types.

Figure S2. Expression box plot showing significant differential expression of FAH in normal vs. tumor tissues

Figure S3. mRNA expression levels of Myc-FAH.

Table S1: Oligonucleotides used for sgRNA plasmid construction.

Table S2: Oligonucleotide sequences used to get PCR amplicon for T7E1 assay.

Table S3: PCR amplicon and cleavage sizes after T7E1 assay.

Figure S4. The original uncropped images for immunoblots in the main figures.

Figure S5. Triplicate images of western blots for significance value.

Figure S1

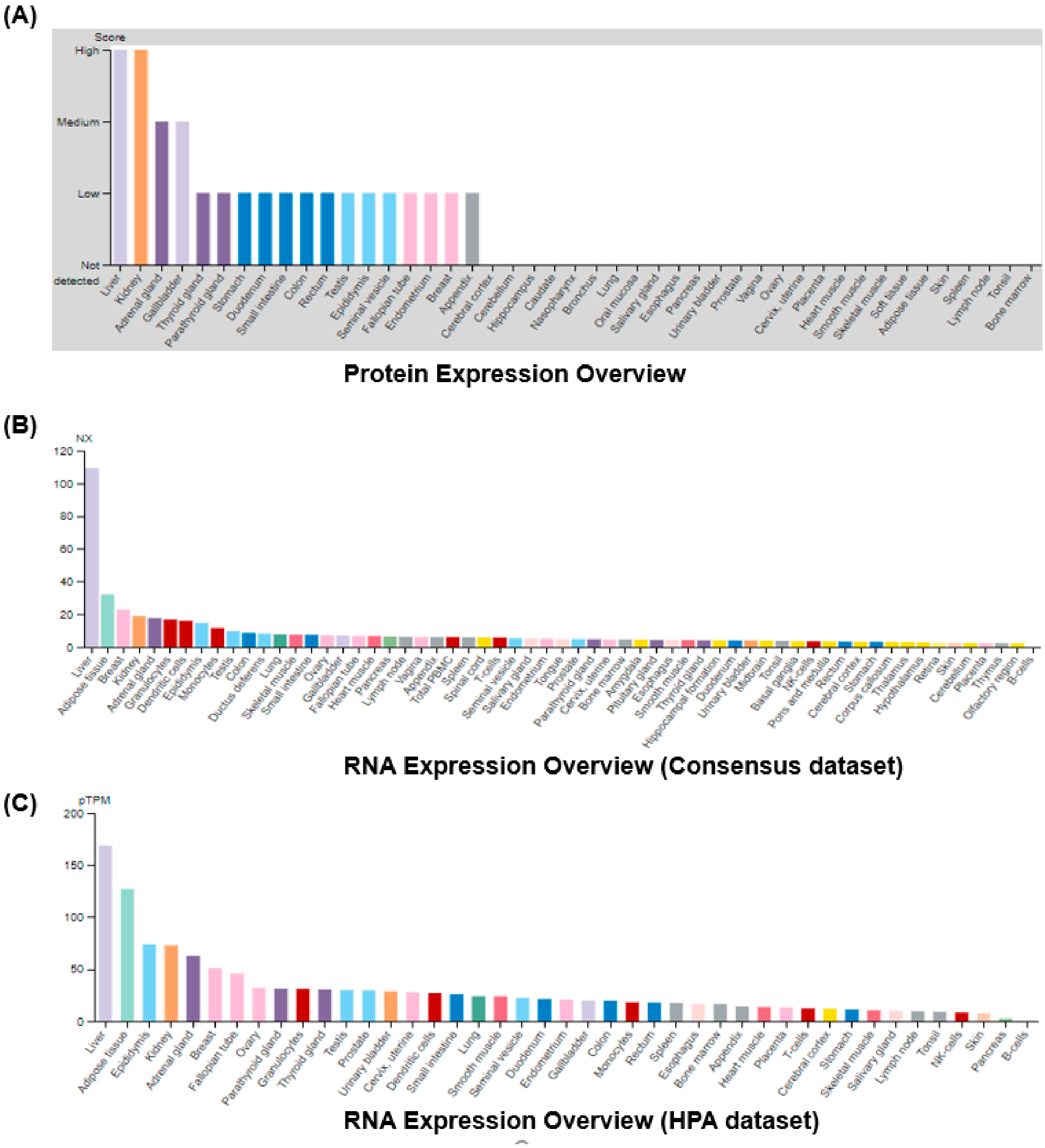
(A)
Score
High
Medium
Low
Not detected
Liver
Kidney
Adrenal gland
Gallbladder
Thyroid gland
Parathyroid gland
Stomach
Duodenum
Small intestine
Colon
Rectum
Testis
Epididymis
Seminal vesicle
Fallopian tube
Endometrium
Breast
Appendix
Cerebral cortex
Cerebellum
Hippocampus
Caudate
Nasopharynx
Bronchus
Lung
Oral mucosa
Salivary gland
Esophagus
Pancreas
Urinary bladder
Prostate
Vagina
Ovary
Cervix, uterine
Placenta
Heart muscle
Smooth muscle
Skeletal muscle
Soft tissue
Adipose tissue
Skin
Spleen
Lymph node
Tonsil
Bone marrow
Protein Expression Overview
(B)
NX
120
100
80
60
40
20
0
Liver
Adipose tissue
Breast
Kidney
Adrenal gland
Granulocytes
Dendritic cells
Epididymis
Monocytes
Testis
Colon
Ductus deferens
Lung
Skeletal muscle
Small intestine
Ovary
Gallbladder
Fallopian tube
Heart muscle
Pancreas
Lymph node
Vagina
Appendix
Total PBMC
Spleen
Spinal cord
T-cells
Seminal vesicle
Salivary gland
Endometrium
Tongue
Prostate
Parathyroid gland
Cervix, uterine
Bone marrow
Amygdala
Pituitary gland
Esophagus
Smooth muscle
Thyroid gland
Hippocampal formation
Duodenum
Urinary bladder
Midbrain
Tonsil
Basal ganglia
NK-cells
Pons and medulla
Rectum
Cerebral cortex
Stomach
Corpus callosum
Thalamus
Hypothalamus
Retina
Skin
Cerebellum
Placenta
Thymus
Olfactory region
B-cells
RNA Expression Overview (Consensus dataset)
(C)
pTPM
200
150
100
50
0
Liver
Adipose tissue
Epididymis
Kidney
Adrenal gland
Breast
Fallopian tube
Ovary
Parathyroid gland
Granulocytes
Thyroid gland
Testis
Prostate
Urinary bladder
Cervix, uterine
Dendritic cells
Small intestine
Lung
Smooth muscle
Seminal vesicle
Duodenum
Endometrium
Gallbladder
Colon
Monocytes
Rectum
Spleen
Esophagus
Bone marrow
Appendix
Heart muscle
Placenta
T-cells
Cerebral cortex
Stomach
Skeletal muscle
Salivary gland
Lymph node
Tonsil
NK-cells
Skin
Pancreas
B-cells
RNA Expression Overview (HPA dataset)

(D)

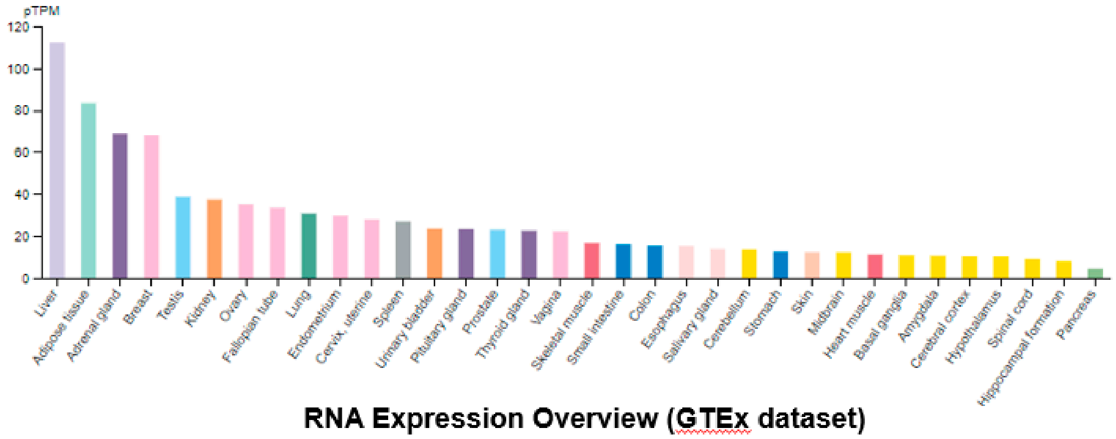

RNA Expression Overview (GTEx dataset)

(E)

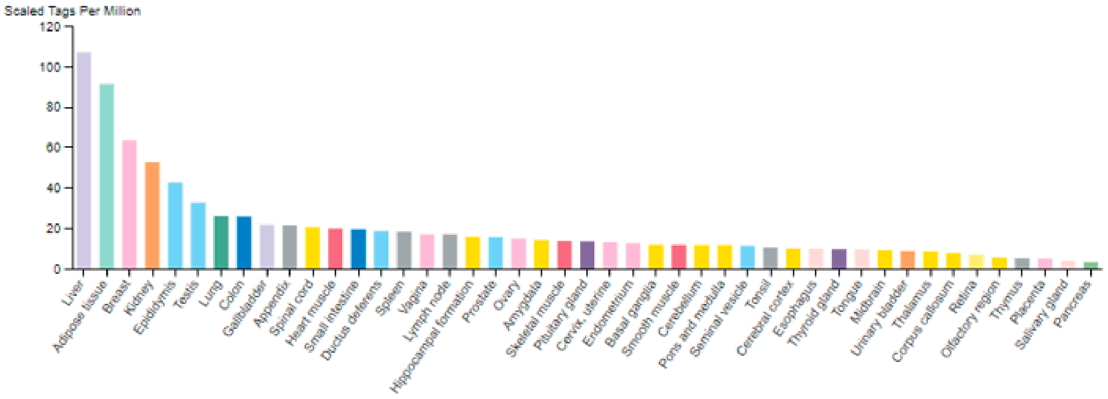

RNA Expression Overview (FANTOM5 dataset)

Figure S1. (A) Comparison of FAH protein expression in different tissue types obtained from The Human Protein Atlas. Comparison of mRNA expression of FAH in different tissue types obtained from (B) Consensus dataset (C) HPA dataset (D) GTEx dataset (E) FANTOM5 dataset.

Figure S2

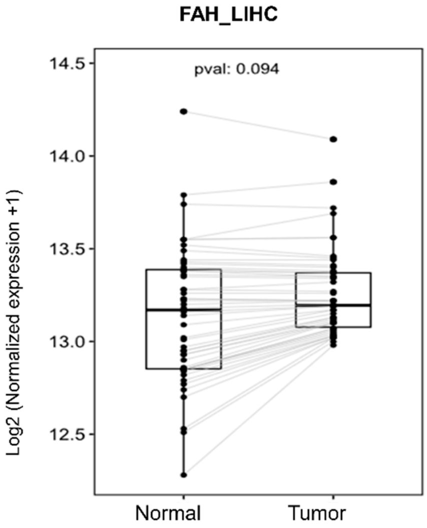

Figure S2. Expression box plot showing significant differential expression of FAH in normal vs. tumor tissues using normal-tumor matched liver cancer patient RNAseq data from TCGA. P value < 0.05 was considered to be statistically significant

Figure S3

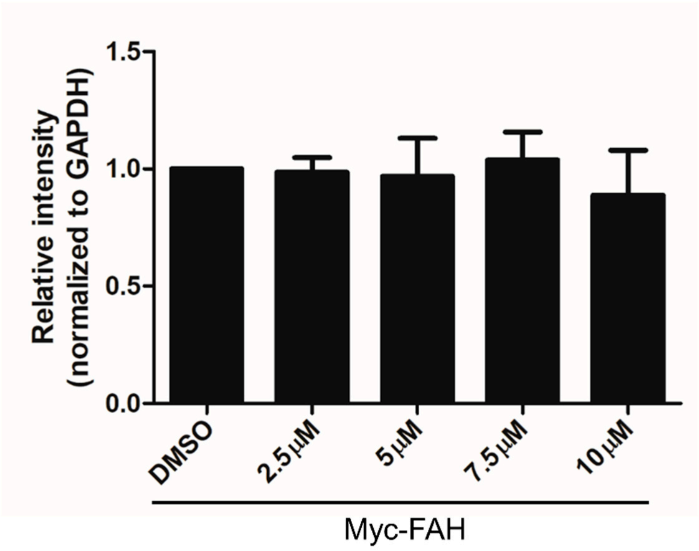

Figure S3: mRNA expression levels of Myc-FAH. Reverse transcription-quantitative PCR was used to assess the mRNA expression levels of Myc-FAH when HEK293 cells are treated with MG132 in a dose-dependent manner.

Table S1: Oligonucleotides used for sgRNA plasmid construction.

Gene	sgRNA	Direction	Sequence (5' to 3')
Cdh1	sgRNA1	FP	GCAGTACACGGAGCACCTGG
		RP	CCAGGTGCTCCGTGTACTGC
	sgRNA2	FP	CGCTTCTGGAACACGCTGAC
		RP	GTCAGCGTGTTCCAGAAGCG

Table S2: Oligonucleotide sequences used to get PCR amplicon for T7E1 assay.

Gene	sgRNA	Direction	Sequence (5' to 3')
Cdh1	sgRNA1 and sgRNA2	FP	AACGACAACAAGGTACCCCC
		FP1	GCTGCTGGTCTGGAATCACT
		RP	CTATGCCTGCTGCCTCACAT
		RP1	CCATCTCCCTTCAGAGCGAC

Table S3: PCR amplicon and cleavage sizes after T7E1 assay.

Gene	sgRNA	PCR size	Cleavage size	Orientation
Cdh1	sgRNA1	355	299+56	Sense
	sgRNA2	355	151+196	Sense

Figure S4: The original uncropped images for immunoblots in the main figures.

Fig 2A

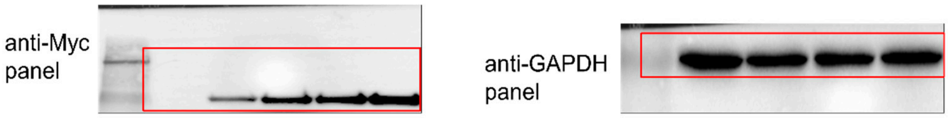

Fig 2B

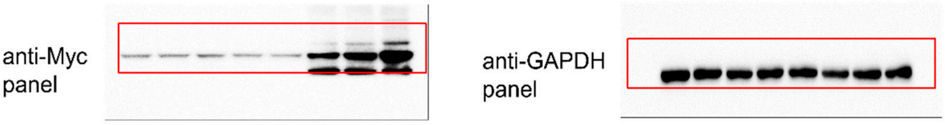

Fig 2C

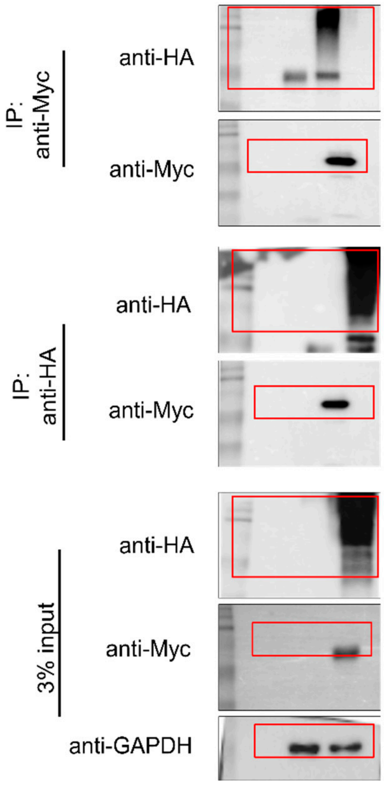

Fig 2D

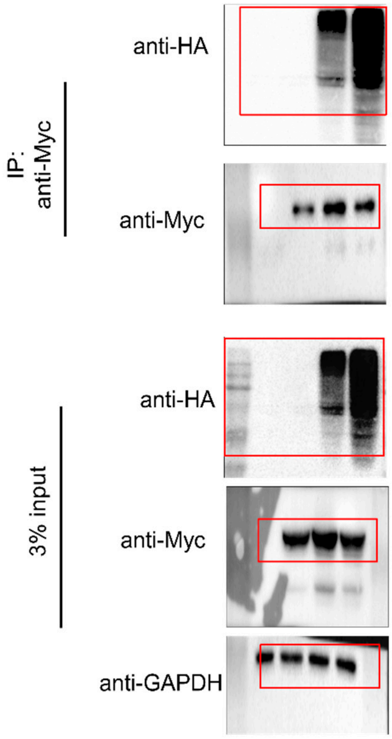

Fig 2E

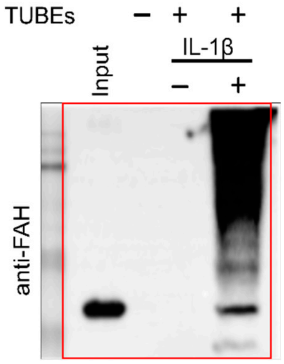

Fig 3A

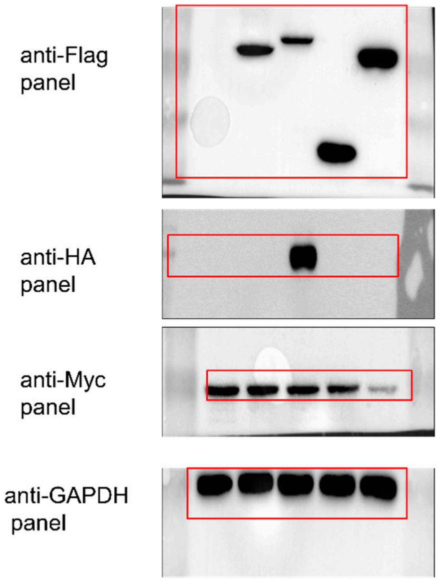

Fig 3D

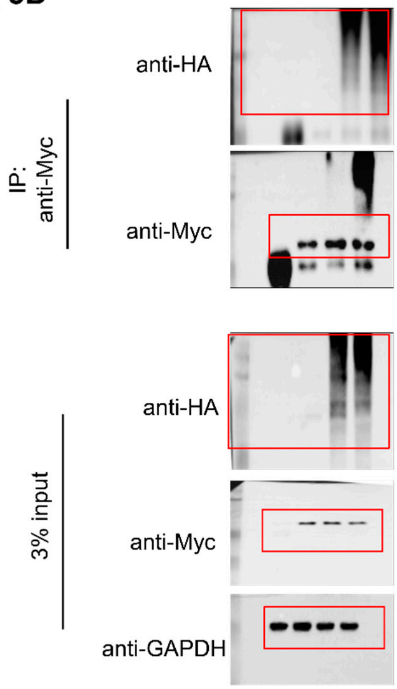

Fig 3B

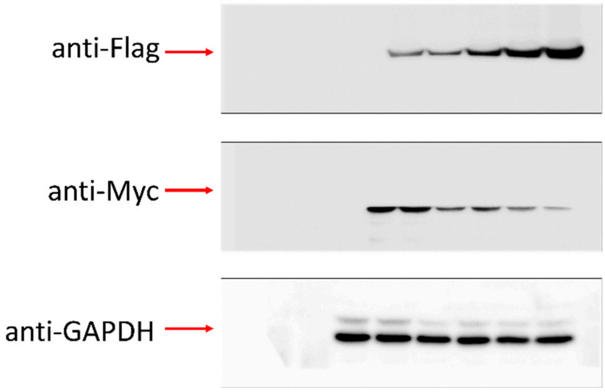

Fig 3C

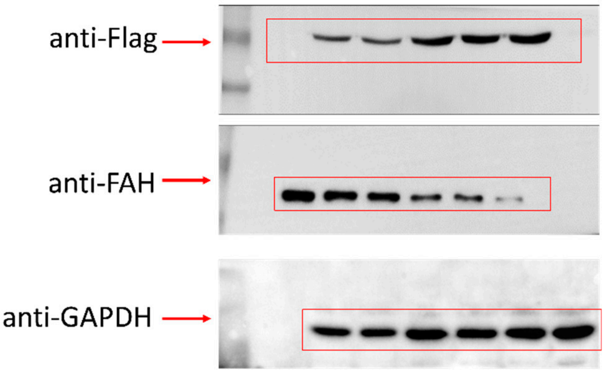

Fig 4C

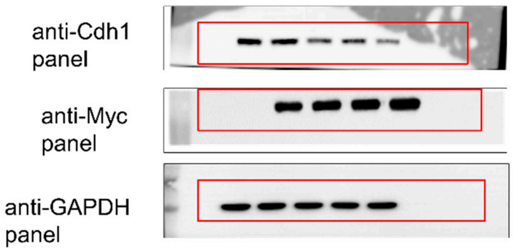

Fig 4D

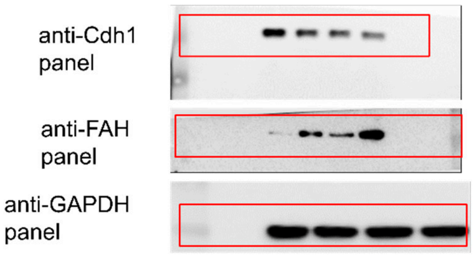

Fig 4E

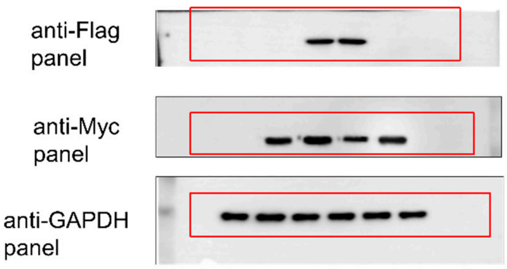

Fig 4F

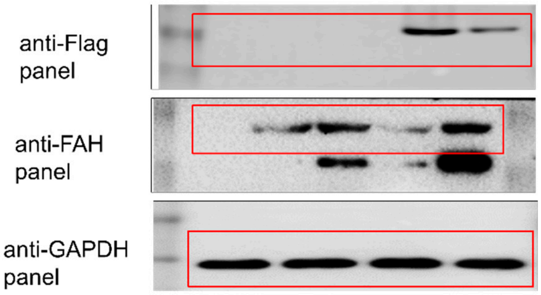

Figure 5

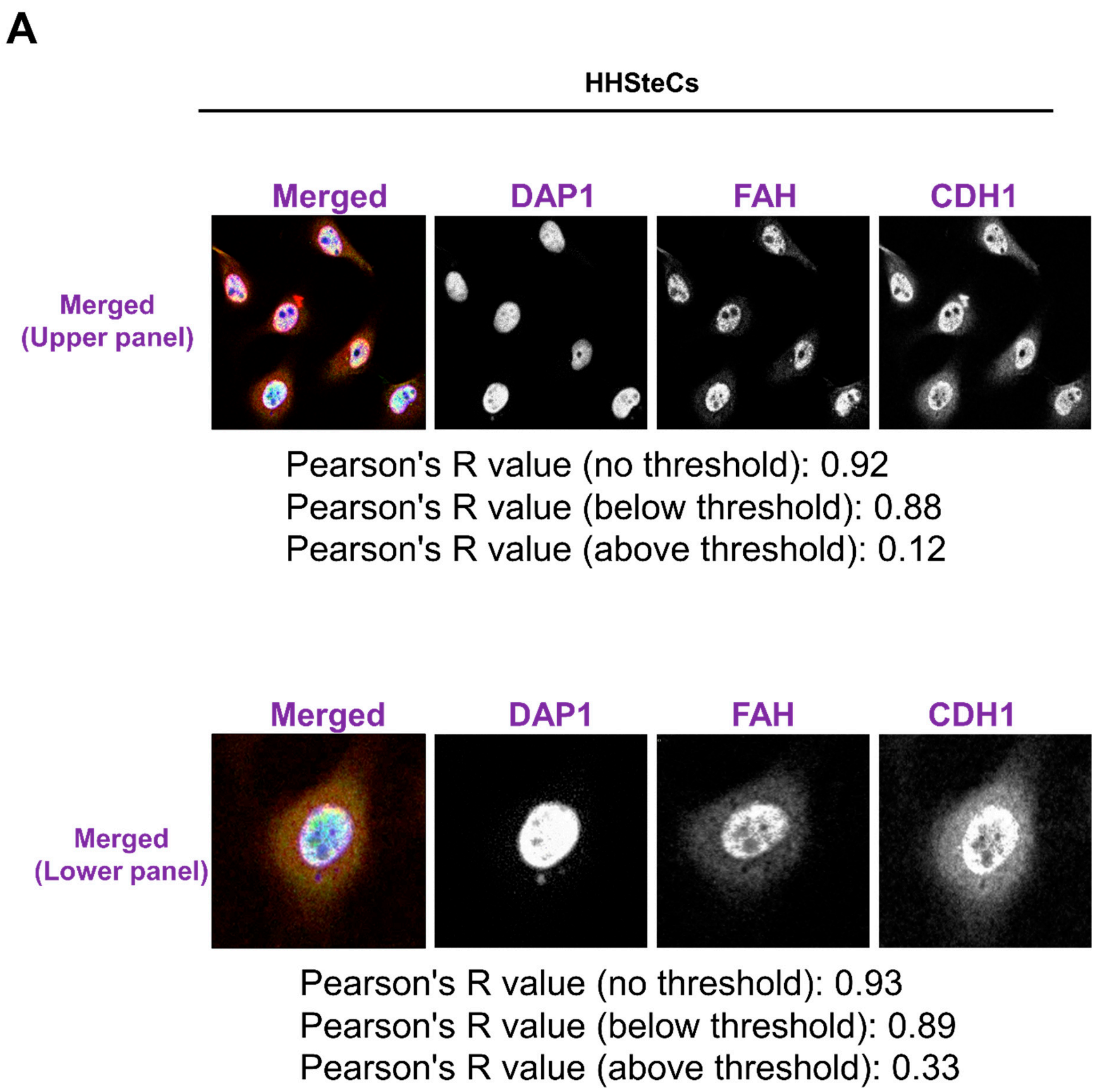
A
HHSteCs
Merged
DAP1
FAH
CDH1
Merged
(Upper panel)
Pearson's R value (no threshold): 0.92
Pearson's R value (below threshold): 0.88
Pearson's R value (above threshold): 0.12
Merged
DAP1
FAH
CDH1
Merged
(Lower panel)
Pearson's R value (no threshold): 0.93
Pearson's R value (below threshold): 0.89
Pearson's R value (above threshold): 0.33

Fig 5B

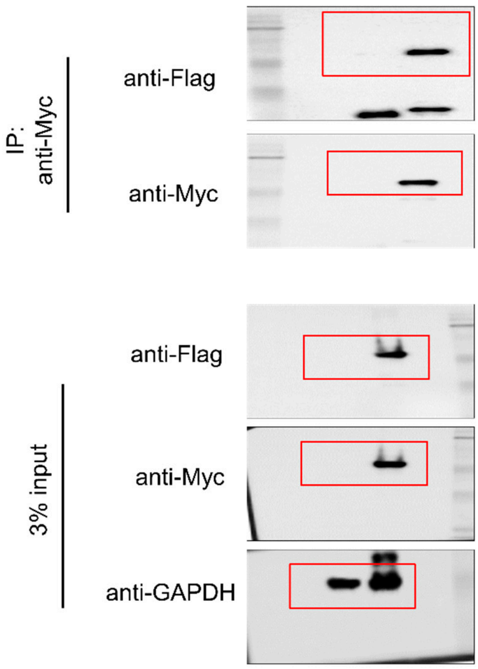

Fig 5C

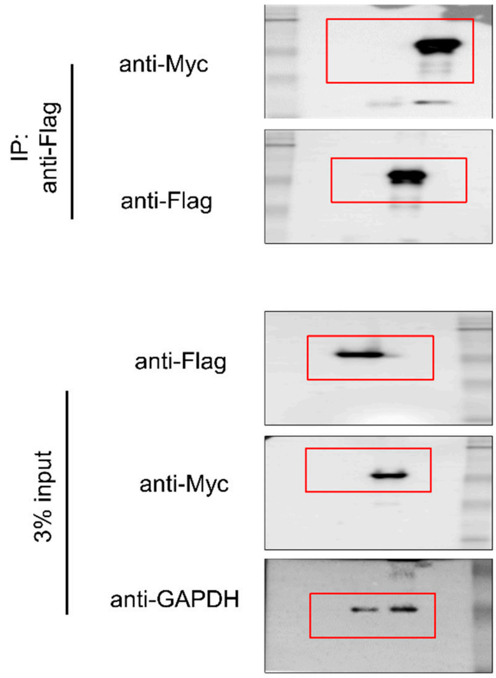

Fig 5D (upper panel)

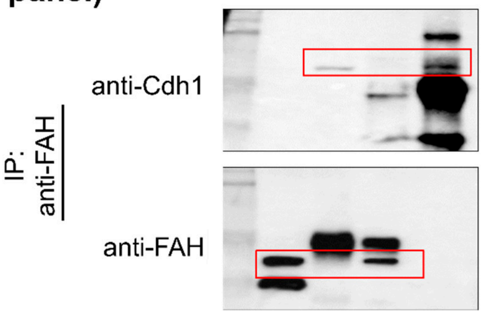

Fig 5D (lower panel)

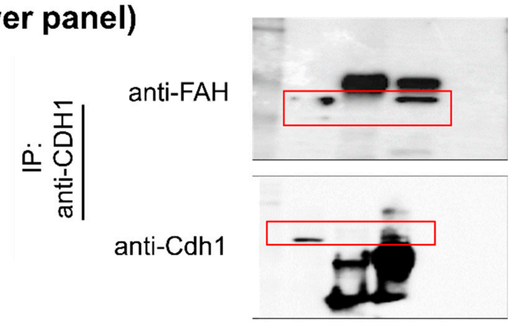

Fig 6A

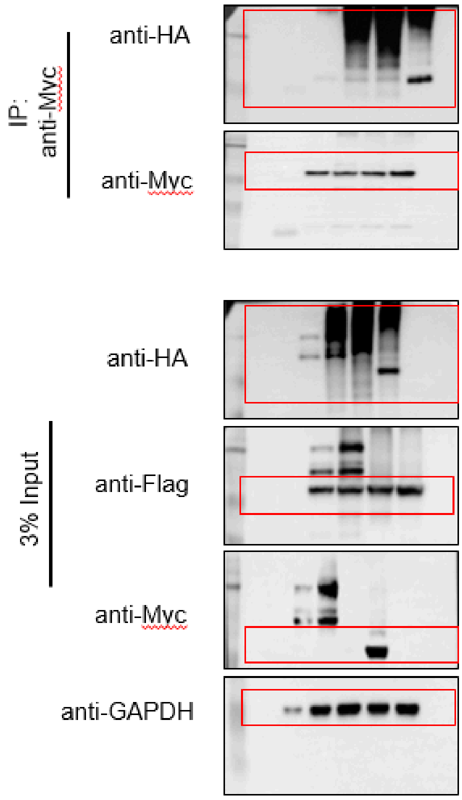

Fig 6B

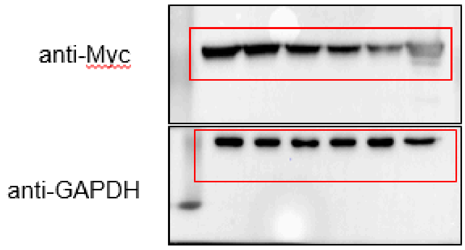

Fig 6C

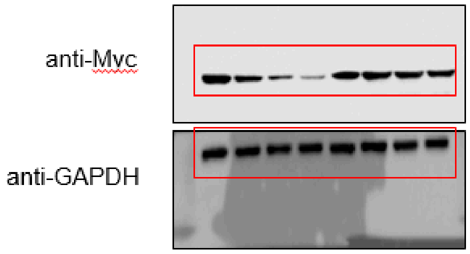

Fig 6D

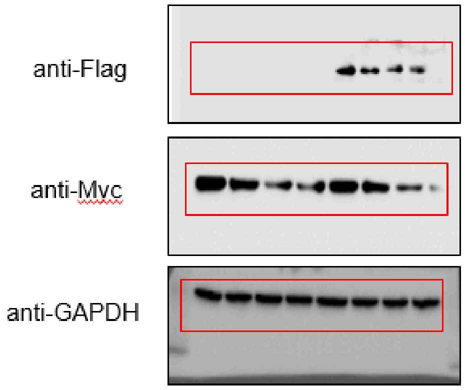

Figure S5. Triplicate images of western blots for significance value.

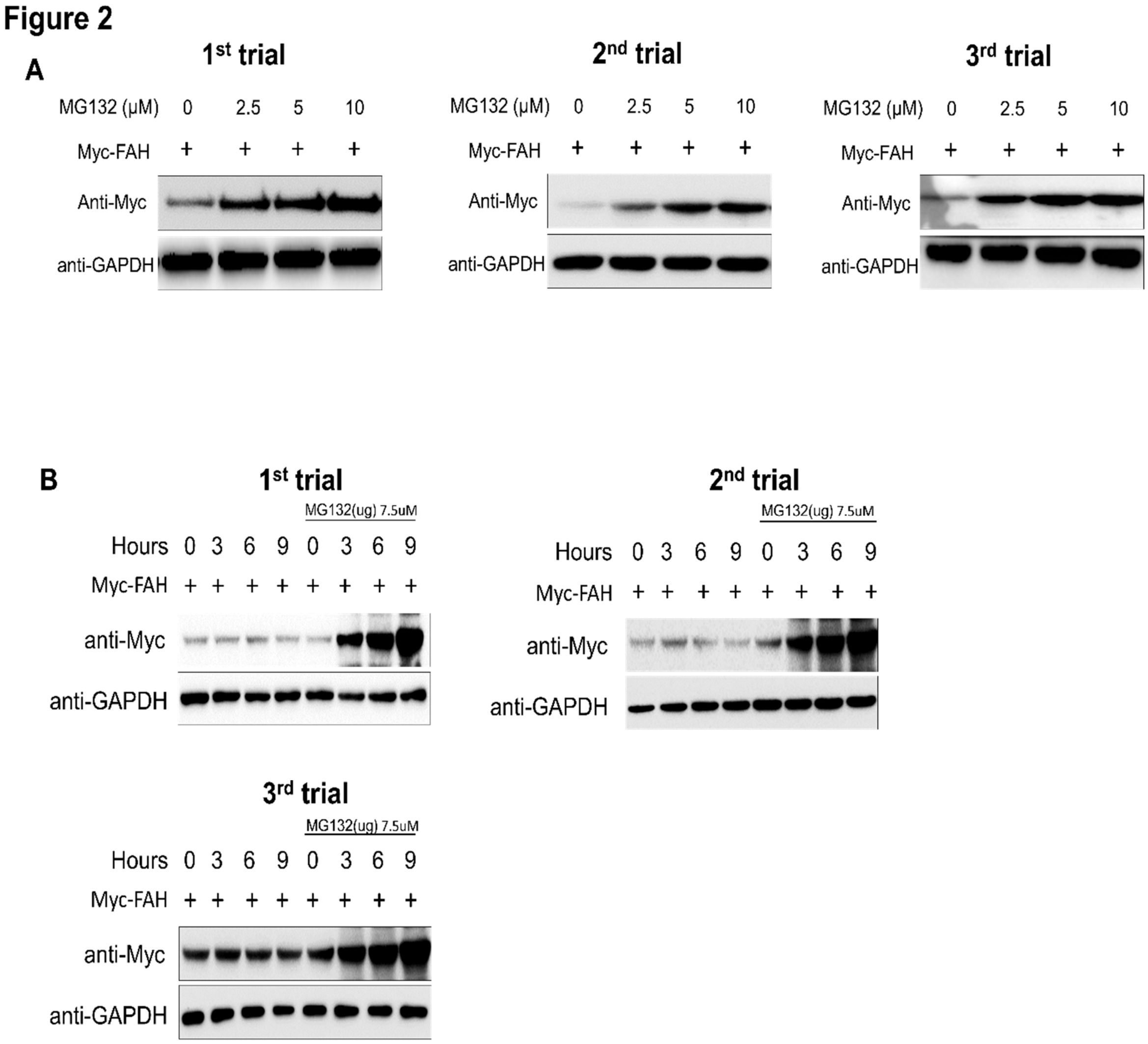

Figure 3

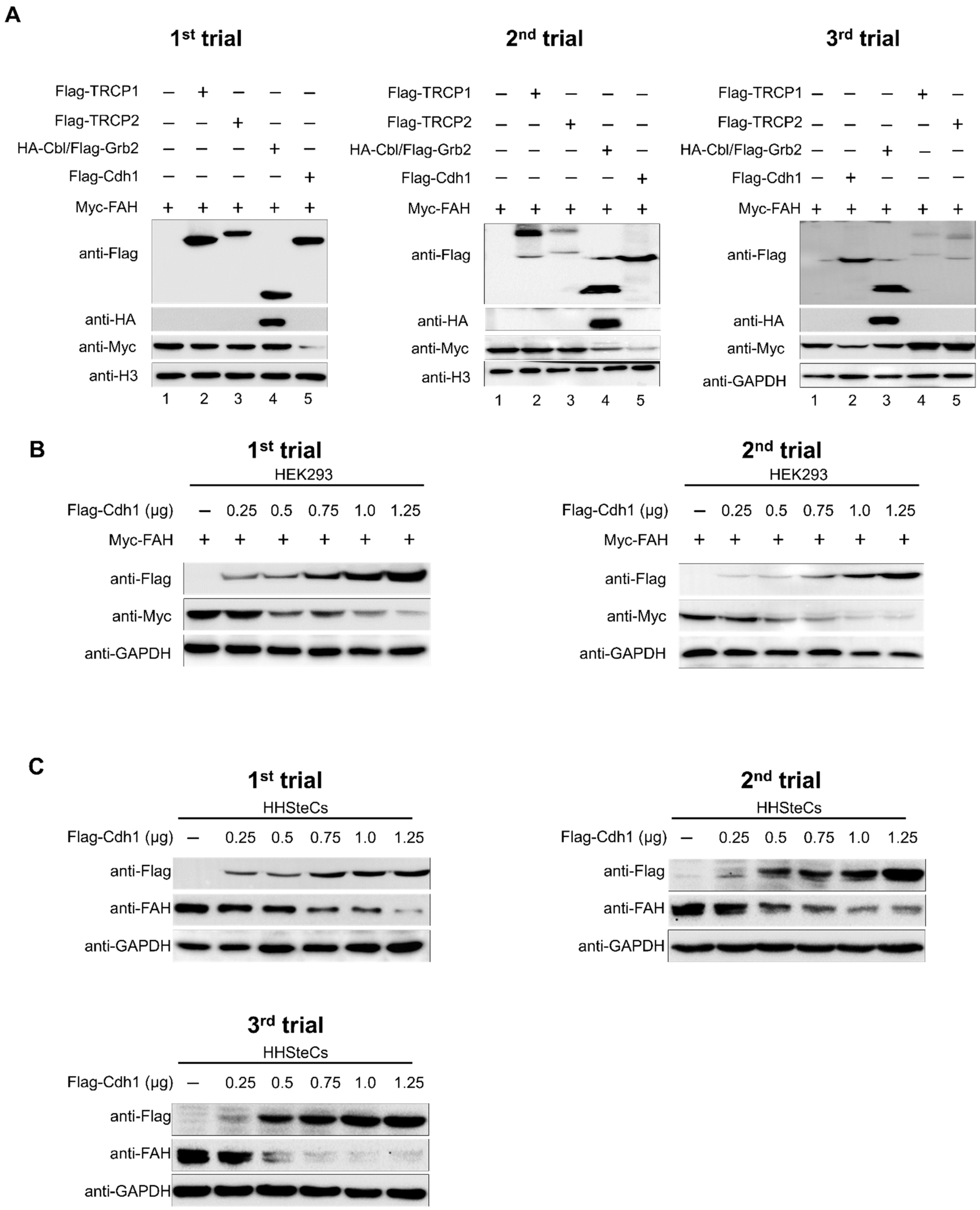
A
1st trial
2nd trial
3rd trial
Flag-TRCP1
Flag-TRCP2
HA-Cbl/Flag-Grb2
Flag-Cdh1
Myc-FAH
anti-Flag
anti-HA
anti-Myc
anti-H3
anti-GAPDH
1 2 3 4 5
B
1st trial
2nd trial
HEK293
Flag-Cdh1 (μg) — 0.25 0.5 0.75 1.0 1.25
Myc-FAH + + + + + +
anti-Flag
anti-Myc
anti-GAPDH
C
1st trial
2nd trial
3rd trial
HHSteCs
Flag-Cdh1 (μg) — 0.25 0.5 0.75 1.0 1.25
anti-Flag
anti-FAH
anti-GAPDH

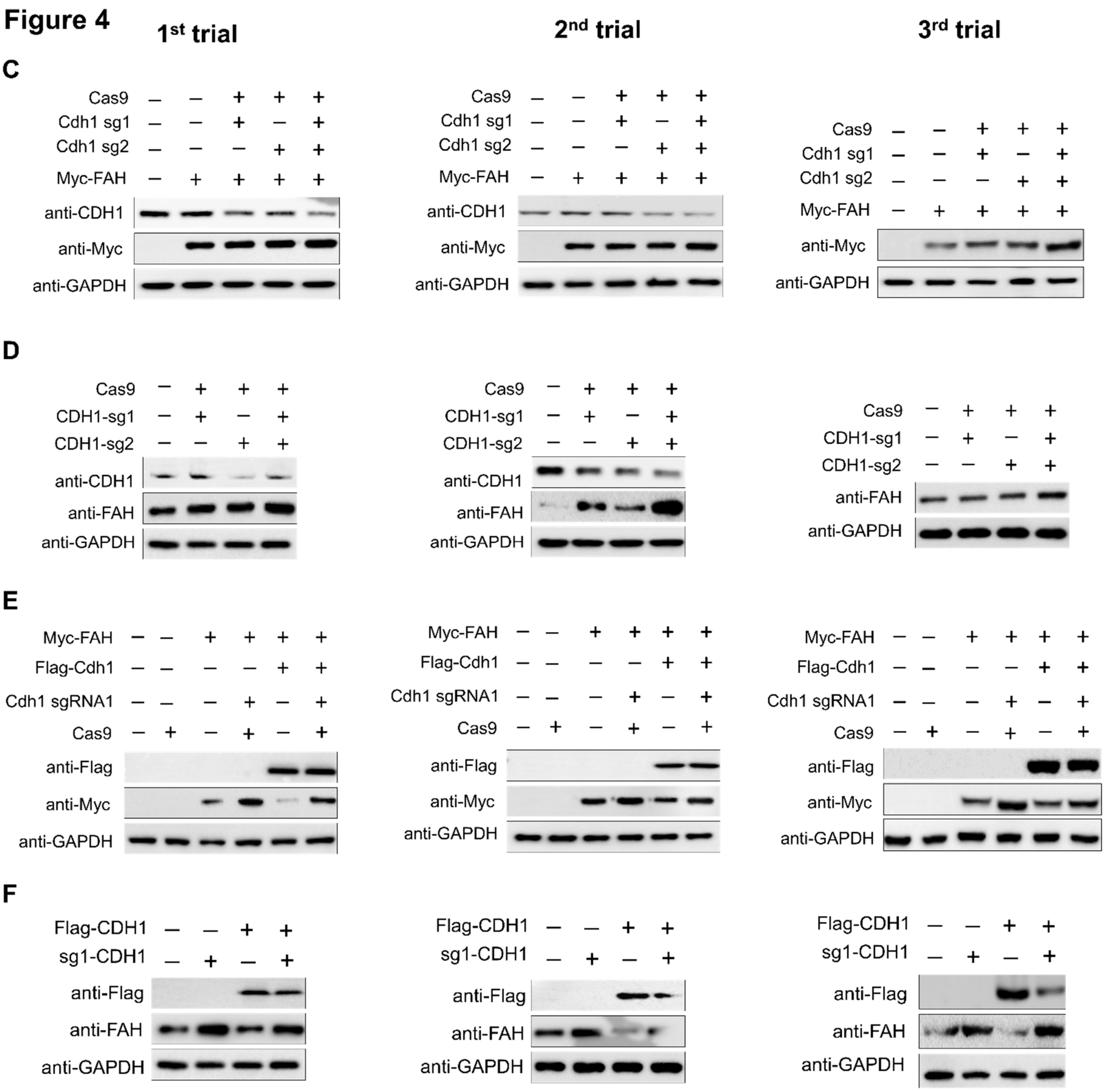
Figure 4
1st trial
2nd trial
3rd trial
C
Cas9
Cdh1 sg1
Cdh1 sg2
Myc-FAH
anti-CDH1
anti-Myc
anti-GAPDH
D
CDH1-sg1
CDH1-sg2
anti-FAH
E
Flag-Cdh1
Cdh1 sgRNA1
anti-Flag
F
Flag-CDH1
sg1-CDH1

Figure 6

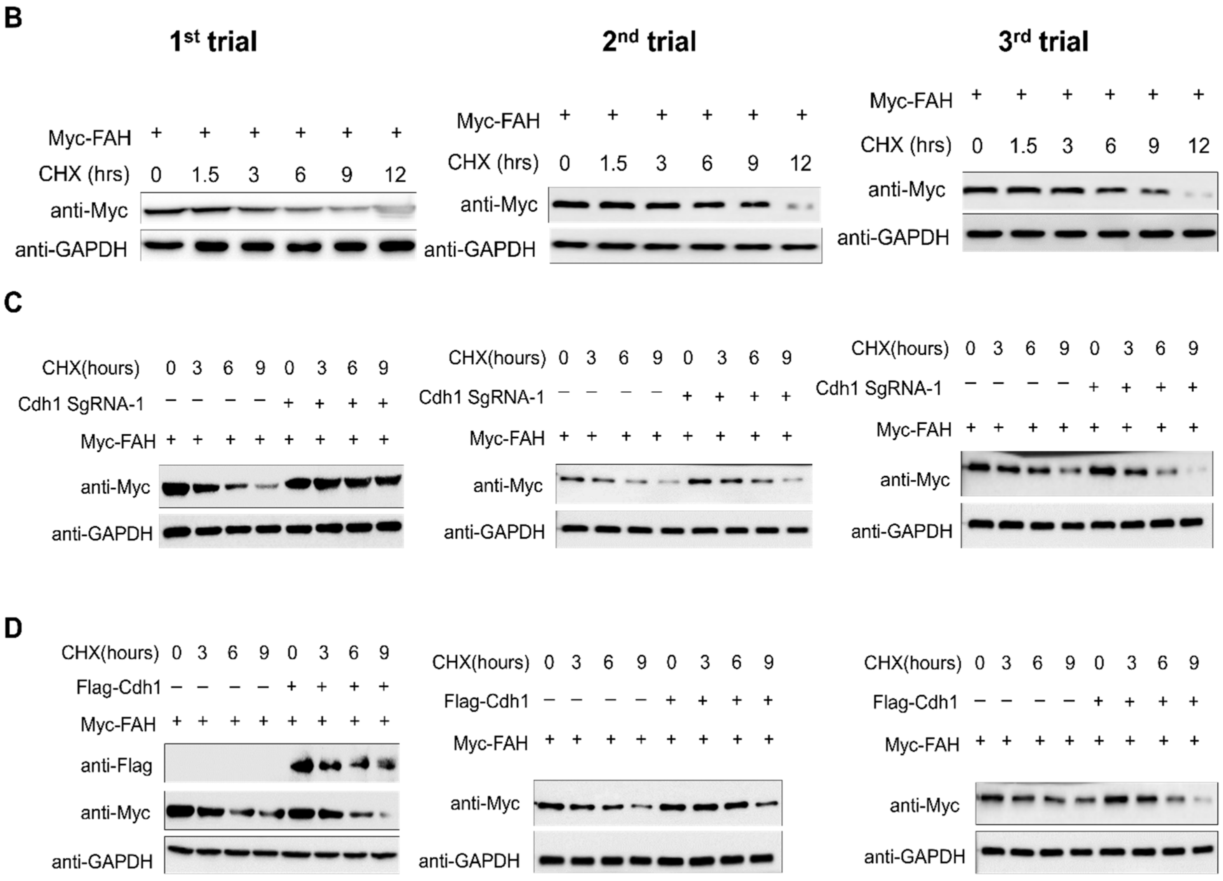
B
1st trial
2nd trial
3rd trial
Myc-FAH
CHX (hrs) 0 1.5 3 6 9 12
anti-Myc
anti-GAPDH
C
CHX(hours) 0 3 6 9 0 3 6 9
Cdh1 SgRNA-1
Myc-FAH
anti-Myc
anti-GAPDH
D
CHX(hours) 0 3 6 9 0 3 6 9
Flag-Cdh1
Myc-FAH
anti-Flag
anti-Myc
anti-GAPDH
